# Structural Evolution of Polyaluminocarbosilane during the Polymer–Ceramic Conversion Process

**DOI:** 10.3390/ma16114172

**Published:** 2023-06-03

**Authors:** Fucheng Xie, Yangpeng Duan, Gaoming Mo, Qing Huang, Zhengren Huang

**Affiliations:** 1 Engineering Laboratory of Advanced Energy Materials, Ningbo Institute of Materials Technology and Engineering, Chinese Academy of Sciences, Ningbo 315201, China; xiefucheng@nimte.ac.cn (F.X.); duanyangpeng@nimte.ac.cn (Y.D.); huangqing@nimte.ac.cn (Q.H.); huangzhengren@nimte.ac.cn (Z.H.); 2 College of Chemical Engineering, Zhejiang University of Technology, Hangzhou 310000, China; 3 Advanced Energy Science and Technology Guangdong Laboratory, Huizhou 516003, China

**Keywords:** SiC ceramic, polyaluminocarbosilane, thermal pyrolysis, structural evolution

## Abstract

Polyaluminocarbosilane (PACS) is an important precursor for silicon carbide (SiC) fibers and ceramics. The structure of PACS and the oxidative curing, thermal pyrolysis, and sintering effect of Al have already been substantially studied. However, the structural evolution of polyaluminocarbosilane itself during the polymer–ceramic conversion process, especially the changes in the structure forms of Al, are still pending questions. In this study, PACS with a higher Al content is synthesized and the above questions are elaborately investigated by FTIR, NMR, Raman, XPS, XRD, and TEM analyses. It is found that up to 800–900 °C the amorphous SiO_x_C_y_, AlO_x_Si_y_, and free carbon phases are initially formed. With increasing temperature, the SiO_x_C_y_ phase partially separates into SiO_2_ then reacts with free carbon. The AlO_x_Si_y_ phase changes into Al_3_C_4_ and Al_2_O_3_ by reaction with free carbon at around 1100 °C. The complicated reactions between Al_3_C_4_, Al_2_O_3_, and free carbon occur, leading to the formation of the Al_4_O_4_C and Al_2_OC phases at around 1600 °C, which then react with the SiC and free carbon, resulting in the formation of the Al_4_SiC_4_ phase at 1800 °C. The amorphous carbon phase grows with the increasing temperature, which then turns into a crystalline graphitic structure at around 1600 °C. The growth of β-SiC is inhibited by the existence of the Al_4_O_4_C, Al_2_OC, and Al_4_SiC_4_ phases, which also favor the formation of α-SiC at 1600–1800 °C.

## 1. Introduction

Polymer-derived ceramics (PDCs) have been widely utilized in fibers and composites, energy storage materials, porous materials, high-temperature sensors, etc. [1,2,3,4,5,6] due to their high-temperature stability, good high-temperature oxidation and corrosion resistance, and low-temperature processing and designable precursor structure. It is common that for fabricating PDCs, polymer synthesis, curing, pyrolysis, or sinter processes are often needed. The structure and property of the precursor influence the structure and quality of the final PDCs to a great extent.

Polyaluminocarbosilane (PACS) is one of the important precursors for silicon carbide (SiC) fibers, ceramics, SiC_f_/SiC and C_f_/SiC composites, SiC coatings and adhesion agents, etc. PACS is usually prepared by the reactions of silicon resins (such as solid carbonsilane (PCS) [2,7], liquid PCS [8] or polysilacarbosilane(PSCS) [9], polydimethylsilane (PDMS) [10],) with aluminium compounds (such as aluminium(III) acetylacetonate (Al(acac)_3_), aluminium butanoxide (Al(C_4_H_9_O)), and dimethyaluminium chloride ((CH_3_)_2_AlCl) [11]), at high temperatures [12]. The molecular structure of PACS and the spinning rheological properties have been substantially studied. Yang et al. [13] have found that in the molecular structure of PACS derived from PCS, the formed AlO_x_ (x = 4–6) groups are the connection centers of PCS, which mainly consists of six-member Si–C rings. It is accepted that the smaller PCS molecules in PACS are crosslinked mainly by Si–O–Al bonds [6,7,8,9,10,11,12,13]. Increasing the weight percentage of Al will result in an increase in the relative branching degree of the molecular structure and the apparent melt viscosity and a decrease in the spinnability of the precursor [14]. The addition of Al to thePDCs will improve their high-temperature oxidation resistance. An et al. [15] and Wang et al. [16] reported that the addition of Al to SiCN ceramics leads to a low oxidation rate when subjected to oxidation at temperatures higher than 1000 °C; the reason is that the formed Al-doped cristobalite oxide layer has a lower permeability to molecular oxygen. Furthermore, during the polymer–ceramic conversion process of oxidative curing PCS, the SiC_x_O_y_ phase is subjected to thermal decomposition above 1300 °C [17,18,19,20], which would form coarser crystals with releasing CO and SiO gases [21,22] and lead to a rapid decrease in mechanical properties for the final SiC products. However, the addition of Al into PCS will inhibit the occurrence of this phenomenon by increasing the compactness and hindering the β-SiC grain growth of the SiC products. During the sintering process of PACS cured by oxygen, the Al_2_O_3_, Al_4_O_4_C, Al_2_OC, and Al_4_SiC_4_ phases have been inferred to be formed, the formed Al-O and Al-C bonds in these phases enrich in the SiC grain boundaries, the Al-C bonds can enter into the SiC lattice, and the Al-O bonds can react with the surface of the SiC grains and even enter into the SiC grains, which will hinder the grain growth of β-SiC [23,24]. However, the structural change for the chemical environment of Al in the broad temperature range from precursor to ceramics is still a pending question, especially for the PACS precursor itself during the polymer–ceramic conversion process.

In this study, PACS with a higher Al content is synthesized using PSCS and Al (acac)_3_ in an autoclave under inert atmosphere [12]. This precursor is thermally pyrolyzed in argon atmosphere, and the structural evolutions—especially the changes for the structural form of Al during the pyrolysis process—are elaborately investigated by FTIR, NMR, Raman, XPS, XRD, and TEM analyses, which is beneficial for regulating the structure and property of the final product.

## 2. Experimental

### 2.1. Synthesis and Pyrolysis of PACS

The PACS was prepared by the high pressure method [12]. Aluminium acetylacetonate (Al(acac)_3_) and liquid polysilacarbosilane (PSCS) with ratios of 9% were used as raw materials. Al(acac)_3_ were purchased from Alfa Aesar. The PSCS were prepared in our laboratory by the thermal decomposition of polydimethylsilane (PDMS) at above 400 °C under inert atmosphere. The weight-average molecular weights M_w_ and polydispersity M_w_/M_n_, the softening point, and the Al content of the precursor are listed in Table 1.

The PACS precursor was thermally pyrolyzed in the temperature ranges of 300–1800 °C at a heating rate of 5–10 °C min^−1^ in an alumina tube/graphite furnace under argon atmosphere, with holding time of 1 h at each heat treatment temperature. In order to investigate the structural evolution during the pyrolysis process, the pyrolysis residues were characterized by various tests.

### 2.2. Characterization

Fourier transform-infrared (FT-IR) spectra (Nicolet 6700 spectrometer, Waltham, MA, USA) were obtained between 4000 and 400 cm^−1^. The molecular weights of the PACS were tested by the gel permeation chromatography (GPC) (HLC-8320, EcoSEC, Tokyo, Japan) method; tetrahydrofuran (THF) was used as the solvent and eluent, and the results were calibrated by polystyrene standards. The softening point of the PACS was measured on a melting point apparatus (Mettler Toledo MP90, Zurich, Switzerland). Solid-state ^13^C CP/MAS NMR spectra, ^29^Si DD/MAS NMR spectra, and ^27^Al MAS NMR spectra were obtained (Agilent 600 DD2, Santa Clara, CA, USA) at resonance frequencies of 150.72 MHz, 199.13 MHz, and 156.25 MHz, respectively. X-ray photoelectron spectroscopy spectra (XPS) were determined (Axis Ultra DLD, Kratos, Japan) using the binding energy of C 1 s (284.8 eV) as a reference. Raman spectroscopy spectra were recorded (Renishaw inVia Reflex spectrometer, London, England) with a wavelength of 532 nm of the excitation source. The X-ray diffraction (XRD) patterns were obtained (Bruker D8 Advance, Karlsruhe, Germany, Cu-Kα radiation) at λ = 0.154 nm in the 2θ ranges of 10–80°. The apparent mean grain size, *L*, of the SiC crystalline phase was calculated according to the Scherrer equation: *L = Kλ/Dcosθ*, where *K* = 0.89, *D* is the width of the (111) diffraction peak at mid-height. The thermogravimetric (TG) analysis was conducted (PerkinElmer Diamond TG/DTA instrument, Waltham, MA, USA) at a heating rate of 10 °C/min up to 1000 °C under argon atmosphere. The weight ratios of the elements C, O, N, and H were determined using a carbon sulphur analyser (CS844, LECO, St. Joseph, MO, USA), an oxygen nitrogen analyser (EMGA-620 W, HORIBA Jobin Yvon, St. Joseph, MO, USA), and an organic element analyser (Elementar, Langenselbold, Germany), respectively. The Al content was measured by the inductively coupled plasma emission spectrum (ICP) method (Spectro Arcos II spectrometer, Düsseldorf, Germany). Before the measurement, the specimen was heat-treated with molten mixtures of sodium carbonate and boric acid, then dissolved with deionized water. The weight ratio of the Si was obtained by difference subtraction. TEM measurements were conducted (FEI Tecnai F20 device, Waltham, MA, USA) at an acceleration voltage of 200 kV.

## 3. Results and Discussion

### 3.1. Molecular Structure Changes and TG Analysis

**Figure 1:**
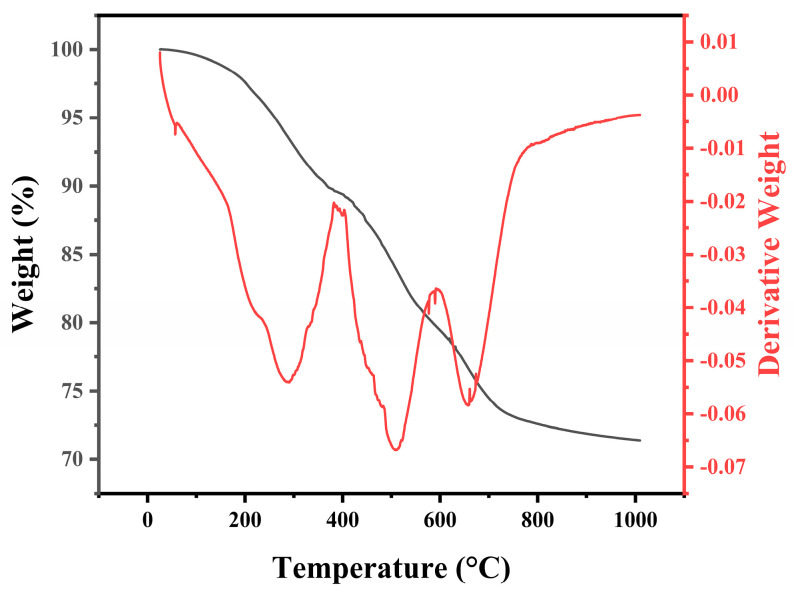
TG and DTG curves as a function of temperature for PACS.

**Figure 2:**
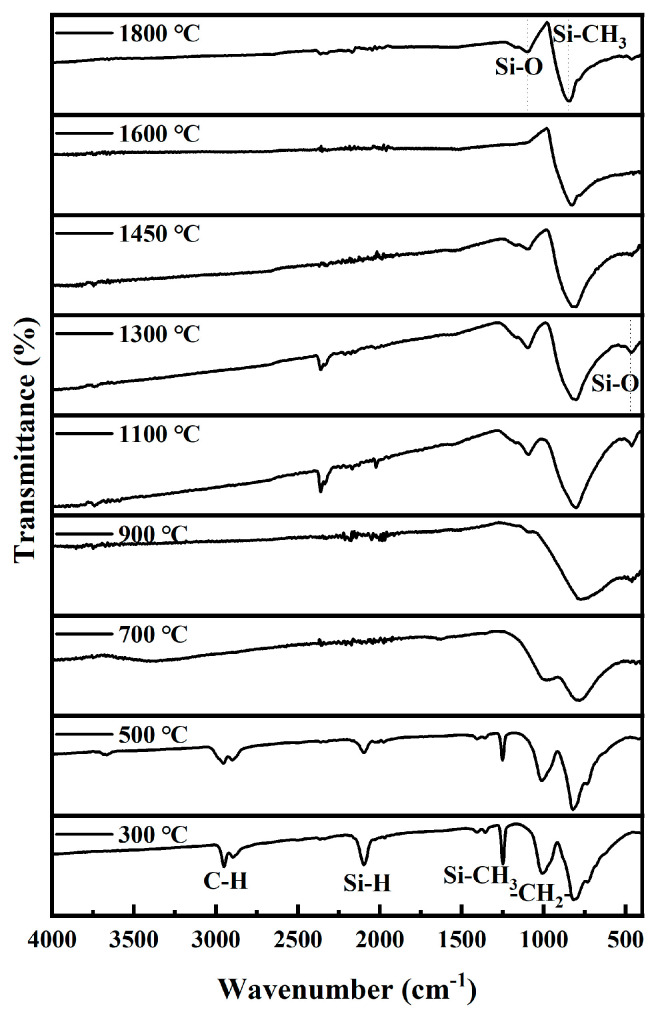
FTIR spectra for pyrolysis residues of PACS at various temperatures.

Figure 1 shows the thermogravimetric (TG) and derivative thermogravimetric (DTG) curves as a function of the temperature in the argon flow. It is observed that the precursor has a ceramic yield of 71.4% at 1000 °C, higher than typical PCS whose ceramic yield is about 60%. The reason is that this precursor has a relatively high Al content and thus more branched and ring molecular structures, leading to a lower weight loss [14]. It is known that the TG curve can be divided into three main stages: (I) up to 400 °C, the weight loss is 10.6%, which is ascribed to the evaporation of the small molecules in the precursor [14,25]. In the DTG curve, the peak at 288 °C may correspond to this weight loss. During this stage, in the FTIR spectra shown in Figure 2, the typical absorption bands observed at 2950 and 2900 cm^−1^, 2100 cm^−1^, 1410 cm^−1^, 1350 cm^−1^, 1250 cm^−1^, 1020 cm^−1^, and 830 cm^−1^ are ascribed to C-H stretching, Si-H stretching of Si-CH_3_, C-H deformation in Si-CH_3_, CH_2_ deformation in Si-CH_2_-Si, Si-CH_3_ deformation, CH_2_ wagging in Si-CH_2_-Si, Si-CH_3_ rocking, and Si-C stretching, respectively. They do not change to any great degree at this stage. The weak bands at 2351 cm^−1^ at 1100 °C and 1300 °C are ascribed to CO_2_. (II) From 400 °C to 800 °C, the weight loss is 16.8%. Similar to PCS, the weight loss stems from thermal decomposition of the precursor molecules. During this stage, decomposition of the side chains and dehydrogenation and dehydrocarbonation condensation reactions occur, accompanied by release of some gases such as H_2_, CH_4_, etc. A three-dimensional network inorganic structure is eventually formed [14,25]. It is reasonable to infer that the dehydrogenation and dehydrocarbonation condensation reactions occur mainly at 400–550 °C and that the decomposition of the side chains occurs mainly at 550–800 °C [25]. Therefore, it is reasonable to infer that the two peaks at 509 °C and 655 °C in the DTG curve correspond to the dehydrogenation and dehydrocarbonation condensation reactions and the decomposition of the side chains, respectively. During this stage, the typical absorption bands for C-H stretching, Si-H stretching, C-H deformation, CH_2_ deformation, Si-CH_3_ deformation, and CH_2_ wagging decrease obviously or even vanish as the temperature increases, with only the broad Si-C stretching band (850 cm^−1^) remaining in the end. (III) From 800 °C to 1000 °C, there is a slight weight loss, because the gas evolution by thermal decomposition in this stage is almost complete. During this stage, there is little characteristic structural change and the pyrolysis residues are still amorphous [14,25]. In the FTIR spectra, two weak bands at about 1100 cm^−1^ and 480 cm^−1^, related to Si-O bonds in the SiO_2_ phase, emerge at temperatures higher than 1000 °C, then diminish with increasing temperature and finally vanish at 1600 °C. This will be explained in the following. 

### 3.2. Structural Form Changes for the Elements of Si, C, and Al

**Figure 3:**
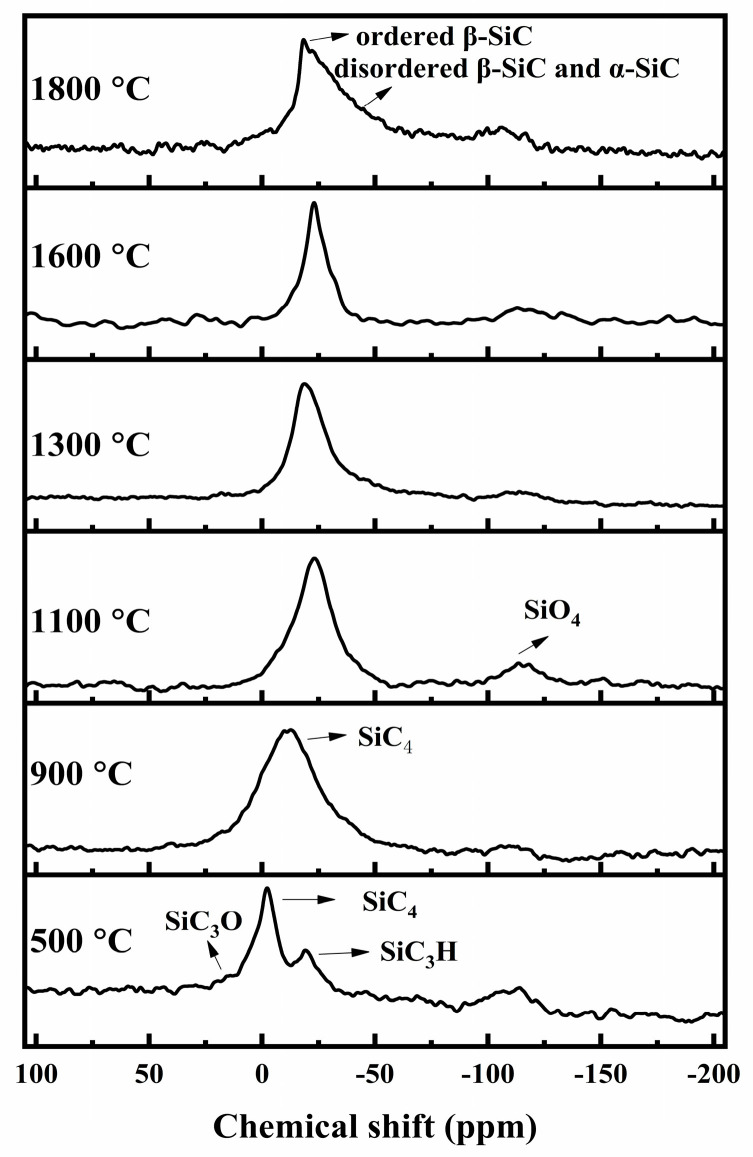
^29^Si MAS NMR spectra for pyrolysis residues of PACS at various temperatures.

**Figure 4:**
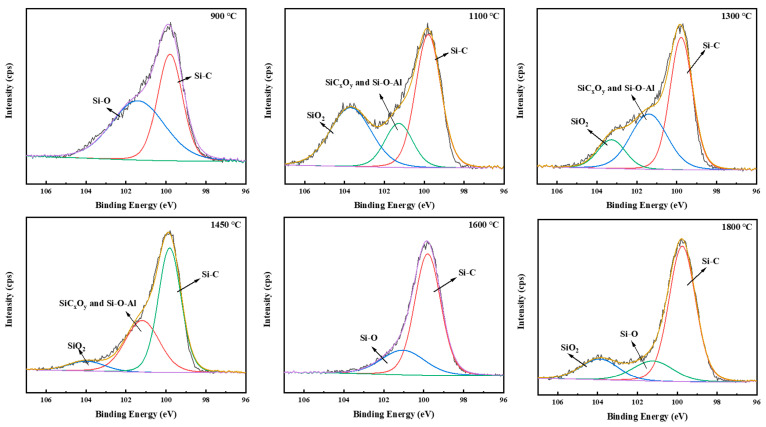
XPS spectra of Si 2p for pyrolysis residues of PACS at various temperatures.

The structural form changes in Si during the pyrolysis process can be expressed by ^29^Si MAS NMR and XPS spectra of Si 2p, as shown in Figure 3 and Figure 4, respectively. In the ^29^Si MAS NMR spectra, at 500 °C, two resonance peaks at −2 ppm and −19 ppm appear, which are attributed to the SiC_4_ and SiC_3_H units as in the PACS precursor [25,26,27]. A weak shoulder peak at 16 ppm is related to the SC_3_O units, which contain Si-O-Si and Si-O-Al bonds [14]. At 900 °C, the broad resonance peak at −12 ppm is ascribed to the amorphous SiC phase [26,27]. As the temperature increases, the broad SiC peak sharpens and shifts upfield, suggesting that the SiC phase becomes more and more ordered and crystallized. A broad-shouldered peak on the right side emerges at 1800 °C, indicating formation of α-SiC and disordered β-SiC in addition to the ordered β-SiC. It is mentioned that a broad resonance peak in the vicinity of −110 ppm is related to the SiO_4_ unit, stemming from SiO_2_ [28] when the precursor is pyrolyzed without air curing. In the XPS spectra of Si 2p, the spectrum at 900 °C can be deconvolved into two main peaks located around 100 eV and 102 eV, which represent the Si-C and Si-O bonds, respectively, although the binding energies of the SiO_2_ and SiO_x_C_y_ phases are a little different [29,30]. The Si-O bonds here mainly come from the SiO_x_C_y_ phase and the Si-O-Al bonds. At 1100 °C, another evident peak at 103.5–104 eV can be deconvolved, which represents the SiO_2_ phase [29,30]. The Si-O bond also has the largest intensity of the FTIR results at this temperature, indicating that the SiO_2_ phase has been formed. SiO_2_ can evolve from the decomposition of the SiO_x_C_y_ phase, shown in Equation (1). The SiO_2_ phase reacts with free carbon with increasing temperature, resulting in SiC, SiO, and CO [28], shown in Equations (2) and (3). Hence the intensity of the SiO_2_ phase decreases at temperatures higher than 1100 °C. Moreover, the SiO_2_ phase can stem from oxygen pollution during the pyrolysis process, as at 1800 °C. At 1600–1800 °C, the Si-O bonds at 101–102 eV may come from the residue Si-O-Si and Si-O-Al bonds in the specimens.

(1) $$SiC_{x}O_{y}\left(s\right)\to SiO_{2}\left(s\right)+SiC\left(s\right)$$

(2) $$SiO_{2}\left(s\right)+C\left(s\right)=SiO\left(g\right)+CO\left(g\right)$$

(3) $$SiO_{2}\left(s\right)+3C\left(s\right)=SiC\left(s\right)+2CO\left(g\right)$$

**Figure 5:**
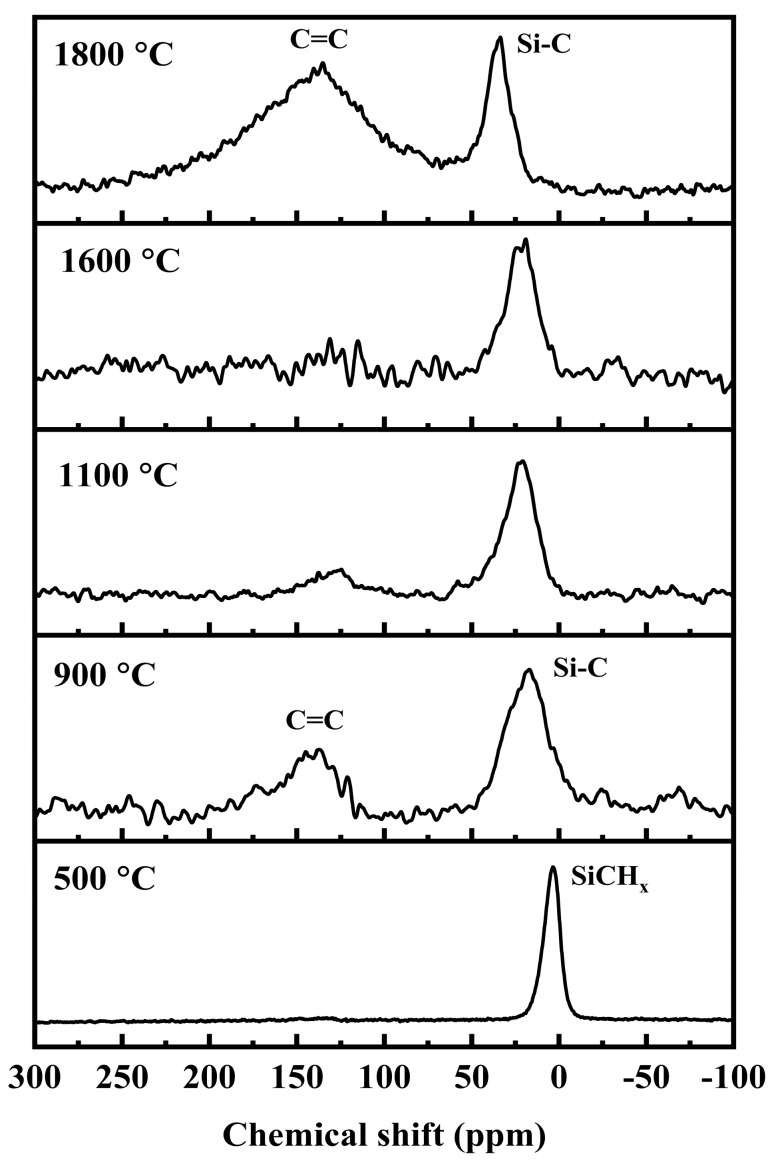
^13^C MAS NMR spectra for pyrolysis residues of PACS at various temperatures.

The ^13^C MAS NMR spectra are shown in Figure 5. In the spectra, the peak at 3 ppm at 500 °C is ascribed to SiCH_x_ (x = 1, 2, 3) groups, which are the same as in the precursors [27,28]. As the temperature increases to 900 °C, a resonance peak at about 16 ppm appears and represents the amorphous SiC phase. The broad SiC peak sharpens and shifts downfield with increasing temperature, also suggesting crystallizing and ordering of the SiC phase. At 900 °C, another weak peak at 130–140 ppm appears, which is due to the C=C bonds of the free carbon phase [26,27,28]. 

**Figure 6:**
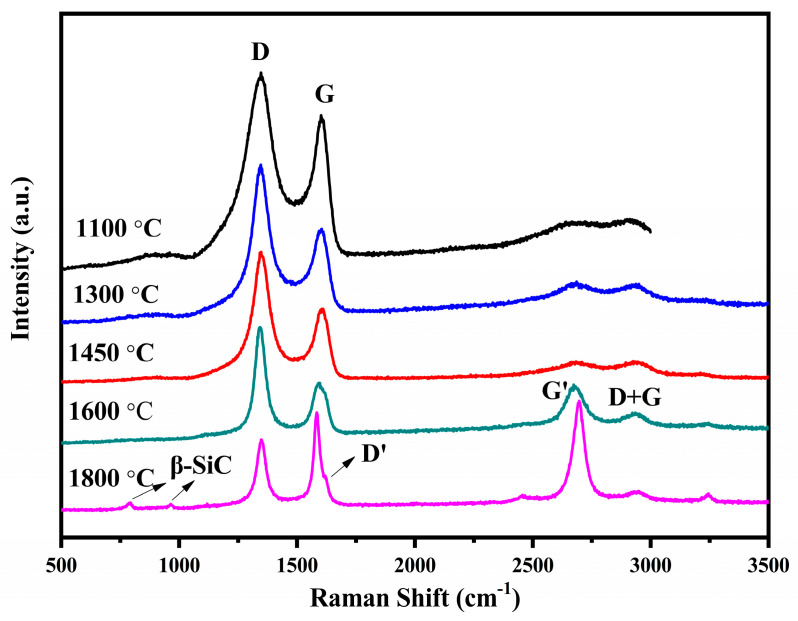
Raman spectra for pyrolysis residues of PACS at various temperatures.

Raman spectroscopy is an effective tool for probing the information of the free carbon phase, which is shown in Figure 6. In the spectra, two peaks at around 1350 cm^−1^ and 1580 cm^−1^ usually appear, called the D band and G band, which typically represent disordering of the free carbon and basal-plane bond stretching of sp^2^ carbon for crystalline graphite, respectively [31,32]. In addition, in the second-order Raman spectra, G’ and D + G bands at approximately 2700 cm^−1^ and 2945 cm^−1^ sometimes appear. Accordingly, the G’ band as an overtone of the D band is commonly found in defect-free graphite specimens. The D + G band can be attributed to a combination of the G and D modes, and is usually found in disturbed graphitic structures [31]. The Raman characteristics are presented in Table 2. With increasing temperature, the G band position shifts toward low wavenumbers, and the intensity ratio of the D and G bands, I_D_/I_G_, increases in the temperature ranges of 1100–1600 °C and then decreases to 1800 °C. From 1600 °C to 1800 °C, the intensity of the G’ band enlarges profoundly. At 1800 °C, a shoulder at 1620 cm^−1^ called the D’ band appears, indicating that crystalline grains of graphitic carbon have been formed. These indicate that at 1600 °C the crystalline graphitic carbon is formed and that the turning point for the free carbon phase changing from amorphous to crystalline is in the temperature range of 1400–1600 °C. From 1100 °C to 1450 °C, with increasing temperature, the number of the ordered C-C rings increases, thus the amorphous carbon becomes more and more turbostratically ordered; therefore, the G band position turns into red-shift and the I_D_/I_G_ increases. With increasing temperature sequentially, the turbostratic free carbon phase continues the growth and rearrangement, leading to formation of graphitic carbon nanocrystallites, which grow again. The graphitic carbon becomes more and more ordered, and therefore the G band position turns into red-shift and the I_D_/I_G_ is decreases to 1600–1800 °C. The size of the amorphous turbostratic carbon clusters and graphitic carbon crystallites, L_a_, can be evaluated by I_D_/I_G_ using the Ferrari–Robertson correlation [33]: $I_{D}/I_{G}=C’(\lambda )\times L_{a}^{2}$, and the TK-correlation [34]: $I_{D}/I_{G}=C(\lambda )/L_{a}$, respectively. The values of L_a_ are calculated according to the above correlations [31]. At 1100–1450 °C, the L^a^ for amorphous turbostratic carbon clusters is calculated by the F-R method and the La for graphitic carbon crystallites is calculated by the TK method at 1600–1800 °C. The results are listed in Table 2. The L_a_ increases along with the increasing temperature, whether it represents the size of the amorphous carbon cluster or the lateral crystallite size of the graphitic carbon.

**Figure 7:**
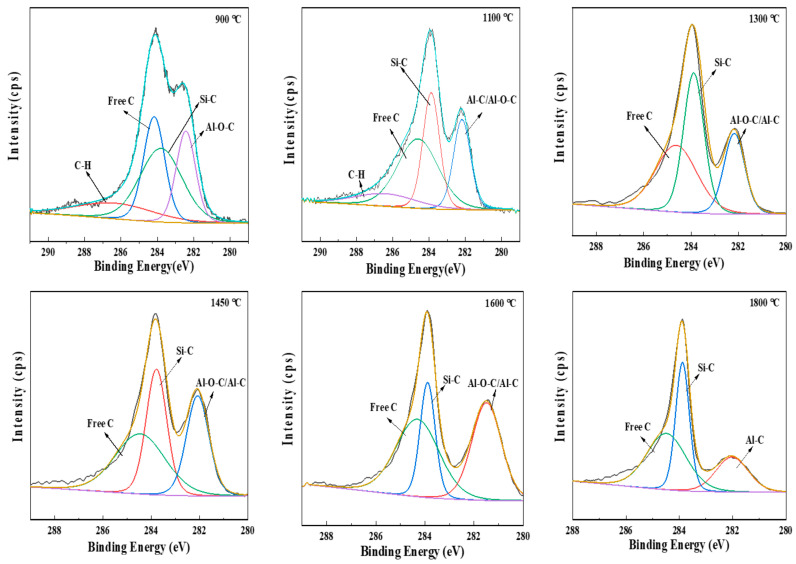
XPS spectra of C 1 s for pyrolysis residues of PACS at various temperatures.

**Figure 8:**
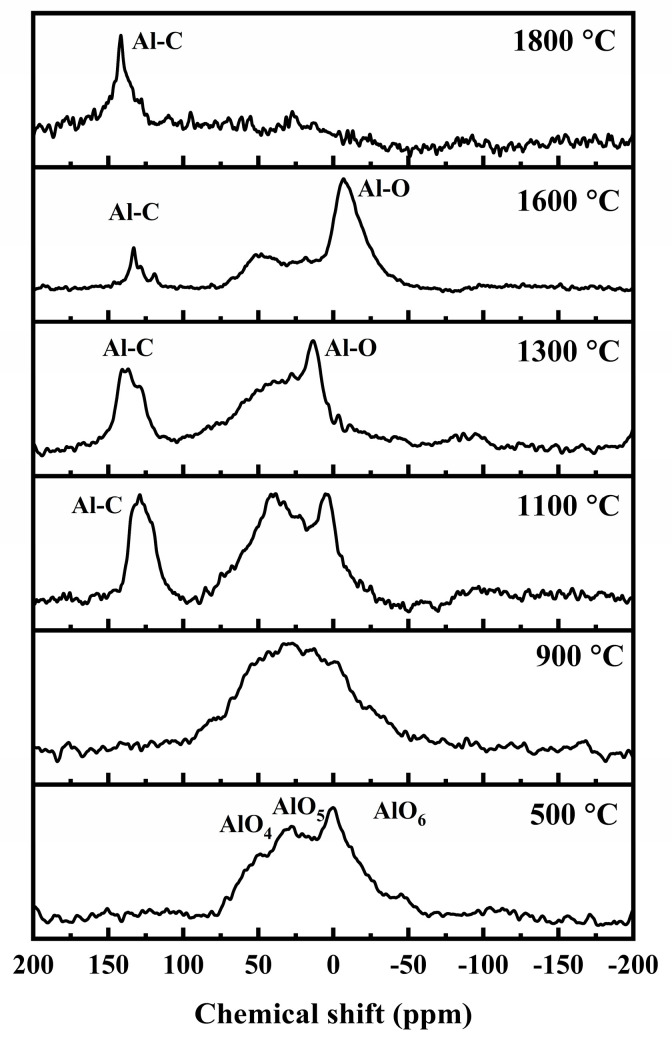
^27^Al MAS NMR spectra for pyrolysis residues of PACS at various temperatures.

The XPS spectra of C 1 s are shown in Figure 7. In a typical XPS spectra of C 1 s for a polymer-derived ceramic (PDC), the C-Si, C=C, and C-C/C-H bonds should be the main chemical bonds with a binding energy of around 283.8, 284.5, and 285.5 eV, respectively. The C-Si bonds (sp^3^ hybridization), the C=C bonds (sp^2^ hybridization), and the C-C bonds (sp^3^ hybridization) are mainly in the SiC-based ceramic matrix, free carbon phase, and aliphatic carbon chains of the precursors, respectively [31]. The C-H bonds (sp^3^ hybridization) are usually present below 1250 °C in the periphery of the free carbon [31]. Accordingly, the peaks for C-Si bonds, the free carbon phase (C=C bonds), and the C-H bonds (below 1250 cm^−1^) are fitted in the spectra as shown in Figure 7. However, there exists another peak located at about 282.2 eV at each temperature which can’t be neglected. The peak is also fitted and ascribed to the C-Al and/or the C-O-Al bonds [35]. To differentiate these bonds more clearly, ^27^Al MAS NMR tests are conducted; the spectra are shown in Figure 8. At 500 °C, three resonance peaks, located at approximately 0, 32, and 55 ppm, are attributed to octahedral AlO_6_, pentacoordinated AlO_5_, and tetrahedral AlO_4_, respectively, as in the PACS precursor [12,13,14]. At 900 °C, these three peaks evolve into a broad peak which is related to amorphous Al-O bonds [18]. The changes in the Al-O bonds suggest that the Si-O-Al bonds may form an amorphous AlO_x_Si_y_ phase by structural rearrangements at 900 °C. The Al-C bonds are not found in the ^27^Al MAS NMR spectrum at 900 °C; therefore, the fitted peak at about 282.2 eV in the XPS spectra of C 1 s can be attributed to the C-O-Al bond at this temperature. The C-O-Al bonds result from the reaction between the Si-O-Al bonds and free carbon, shown in Equation (4). At 1100 °C and 1300 °C, the Al-O bonds split again and an evident peak appears at around 130 ppm, which is ascribed to Al-C bonds [18,19,20,23,24]. In the low temperature range of 1100–1300 °C, the appearance of the Al-C bond can be ascribed to the formation of an Al_4_C_3_ phase; the split of Al-O bonds indicates the formation of the Al_2_O_3_ phase, which both originate from the amorphous AlO_x_Si_y_ phase, as shown in Equation (5). At 1600 °C, the intensity of the Al-C bond decreases while the intensity of the Al-O bonds increases, suggesting the formation of new phase. Based on thermodynamics analysis and experimental research [36,37], Equations (6) and (7) may occur when T ≥ 1560 °C and T ≥ 1259 °C, and Equations (8) and (9) may occur when T ≥ 1620 °C. Therefore, it is reasonable to infer that at temperatures around 1600 °C the Al_4_O_4_C and Al_2_OC phases have been formed by the consumption of Al_4_C_3_, Al_2_O_3_, and free carbon. With an increase in temperature, the Al_4_O_4_C and Al_2_OC phases can be converted into Al_4_SiC_4_ by reactions with SiC and free carbon [38], as shown in Equations (10) and (11). Hence the Al-C bonds increase to a great degree, while the Al-O bonds decease and even vanish at 1800 °C in the ^27^Al MAS NMR spectra.

(4) Si−O−Al+2C=Al−O−C+SiC

(5) $$AlO_{x}{Si}_{y}\left(s\right)+C\left(s\right)\to {Al}_{4}C_{3}\left(s\right)+{Al}_{2}O_{3}\left(s\right)+SiC\left(s\right)+CO\left(g\right)$$

(6) $${Al}_{4}C_{3}\left(s\right)+4{Al}_{2}O_{3}\left(s\right)=3{Al}_{4}O_{4}C\left(s\right)$$

(7) $$2{Al}_{2}O_{3}\left(s\right)+3C\left(s\right)={Al}_{4}O_{4}C\left(s\right)+2CO\left(g\right)$$

(8) $${Al}_{4}C_{3}\left(s\right)+{Al}_{2}O_{3}\left(s\right)=3{Al}_{2}OC\left(s\right)$$

(9) $${Al}_{2}O_{3}\left(s\right)+3C={Al}_{2}OC\left(s\right)+2CO\left(g\right)$$

(10) $${Al}_{4}O_{4}C\left(s\right)+SiC\left(s\right)+6C(s)={Al}_{4}SiC_{4}(s)+4CO\left(g\right)$$

(11) $${2Al}_{2}OC\left(s\right)+SiC\left(s\right)+3C(s)={Al}_{4}SiC_{4}(s)+2CO\left(g\right)$$

### 3.3. Chemical Position and Crystal Structure Changes

The chemical compositions for the PACS and the pyrolysis products at 1300 °C and 1800 °C are listed in Table 3. The PACS precursor is not subjected to oxidative curing; thus the precursor and the pyrolysis residues are all carbon-rich. The decease in oxygen content at above 1300 °C corresponds to the thermal decomposition of the SiO_x_C_y_ phase and the consumption of the AlO_x_Si_y_ phase, as illustrated above. Strangely, the Al content drops largely at 1800 °C. One reason for this is that the Al content is not easily determined precisely at this temperature, and another reason may be the reaction between the SiC and the Al_2_O_3_ at high temperature, with the release of Al_2_O, as shown in Equation (12).

(12) $$SiC\left(s\right)+{Al}_{2}O_{3}\left(s\right)=SiO\left(g\right)+{Al}_{2}O\left(g\right)+CO\left(g\right)$$

**Figure 9:**
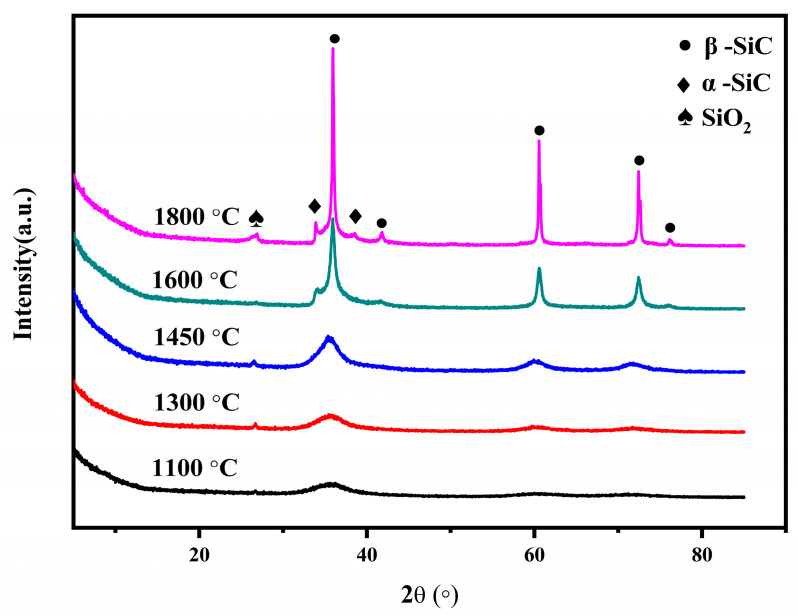
XRD patterns for the pyrolysis residues of PACS at various temperatures.

The XRD patterns for the pyrolysis residues of the PACS at various temperatures are shown in Figure 9. It is seen that at 1100 °C there exists a broad weak peak at 35.7°, suggesting that the residue is still amorphous at this temperature. At 1300 °C, the peaks at 35.7°, 60.5°, and 72.4° emerge distinctly, which are assigned to (111), (220), and (311) diffraction peaks of β-SiC. With increasing temperature, the intensities are increased. At 1600 °C and 1800 °C, the peaks at 34.1° and 38.6° appear, which are assigned to α-SiC. The apparent mean grain sizes of the SiC crystalline phase (111) are calculated by the Scherrer equation, which are 16 nm, 19 nm, 32 nm, and 59 nm at 1300 °C, 1450 °C, 1600 °C, and 1800 °C, respectively. The little weak peak at 26.6° is attributed to SiO_2_, coinciding with the XPS and FTIR results. At high temperatures, the formed Al_4_O_4_C, Al_2_OC, and Al_4_SiC_4_ phases enrich in the SiC grain boundaries and the Al-C bonds in Al_4_SiC_4_ can easily enter into the SiC lattice because of the similar bond length of Al-C (0.195 nm) and Si-C (0.188 nm), which can easily inhibit the SiC grain growth. The Al_4_O_4_C and Al_2_OC phases react with the SiC phase to favor the formation of the α-SiC at the expense of the β-SiC via formation of a solid solution between the phases.

**Figure 10:**
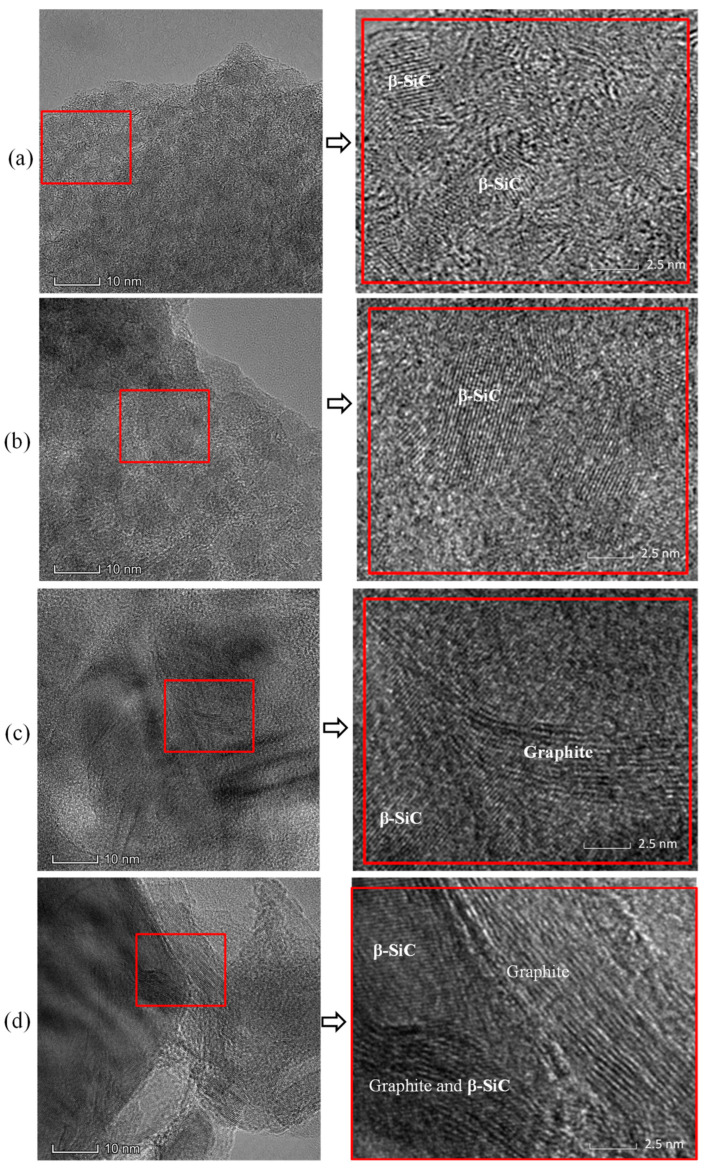
HRTEM images for the pyrolysis residues of PACS at (**a**) 1300 °C, (**b**) 1450 °C, (**c**) 1600 °C, and (**d**) 1800 °C.

The HRTEM images for the pyrolysis residues of the PACS at 1300–1800 °C are presented in Figure 10. The crystalline β-SiC phase is clearly seen, and the grain size enlarges with increasing temperature, corresponding to the XRD results. At 1300 °C and 1450 °C, the graphitic carbon phase is not seen, indicating the free carbon is almost amorphous. As the temperature increases to 1600 °C, the graphitic carbon phase can be clearly observed, and the size enlarges with increasing temperature. The results coincide with the Raman analysis.

## 4. Conclusions

In this study, PACS with a higher Al content is synthesized and the structural evolutions as well as the changes for the structural form of the elements of Si, C, and Al during the pyrolysis process are elaborately investigated. It is found that the polymer turns into a three-dimensional network inorganic structure up to 800–900 °C by dehydrogenation and dehydrocarbonation condensation reactions (400–550 °C) and decomposition of the side chains (550–800 °C). The amorphous SiO_x_C_y_, AlO_x_Si_y_, and free carbon phase are initially formed. From 800 °C to 1200 °C, the pyrolysis products are mainly amorphous, but the SiO_x_C_y_ phase partially separates into SiO_2_, and the AlO_x_Si_y_ phase turns into Al_3_C_4_ and Al_2_O_3_ by reaction with free carbon. Amorphous turbostratic carbon is formed, and the cluster size enlarges with the increasing temperature. From 1200 °C to 1600 °C, the formed SiO_2_ reacts with free carbon with the formation of SiC and the release of SiO and CO. The crystalline β-SiC phase is formed, and the grain size enlarges with the increasing temperature. Complicated reactions between the Al_3_C_4_, Al_2_O_3_, and free carbon occur, leading to the formation of an Al_4_O_4_C and Al_2_OC phase at around 1600 °C. The amorphous turbostratic carbon phase continues to grow, and it changes into a crystalline graphitic structure at around 1600 °C. From 1600 °C to 1800 °C, the β-SiC and crystalline graphitic carbon phases continue to grow. The Al_4_O_4_C and Al_2_OC phases are gradually converted into Al_4_SiC_4_ by reactions with the SiC and free carbon, which favors the formation of α-SiC via formation of a solid solution between the phases. Moreover, the SiC grain growth is inhibited because of the Al_4_O_4_C, Al_2_OC, and Al_4_SiC_4_ phases enriching in the SiC grain boundaries and the Al-C bonds in the Al_4_SiC_4_ entering into the SiC lattice. In addition, the Al element is partially lost by the evaporation of the formed Al_2_O gas. These new discoveries may provide theoretical references for regulating the structures and properties of the final product, such as fibers, bulk ceramics, membranes, foams, etc.

## Figures and Tables

**Table 1:** Molecular weights and polydispersity, softening point, and Al content of PACS.

Sample	Mw	Mn	Mw/Mn	Softening Point (°C)	Al Content (wt%)
PACS-3	3150	1079	2.92	220.7	1.24

**Table 2:** Raman characteristics for the pyrolysis residues of PACS at various temperatures.

Sample	G Band Position (cm^−1^)	I_D_/I_G_	La (nm)
PACS-1100	1606	1.25	1.5
PACS-1300	1605	1.57	1.7
PACS-1450	1602	1.72	1.77
PACS-1600	1593	1.81	2.74
PACS-1800	1583	0.75	6.61

**Table 3:** Chemical compositions for PACS and the pyrolysis residues at typical temperatures.

	Si (wt%)	C (wt%)	O (wt%)	Al (wt%)	nC/nSi
PACS	47.69	38.3	4.85	1.24	1.87
PACS-1300	55.75	34	8.24	2.01	1.42
PACS-1800	64.41	33.9	1.12	0.57	1.23

## Data Availability

The data presented in this study are available on request from the corresponding author.

## References

[1] Yajima S., Hayashi J., Omori M., Okamur K. (1976). Development of a silicon carbide fibre with high tensile strength. Nature.

[2] Ishikawa T., Kohtoku Y., Kumagawa K., Yamamura T., Nagasawa T. (1998). High-strength alkali-resistant sintered SiC fibre stable to 2200 °C. Nature.

[3] Riedel R., Kleebe H., Schönfelder H., Aldinger F. (1995). A covalent micro/nano- composite resistant to high-temperature oxidation. Nature.

[4] Chen Y., Li C., Wang Y., Zhang Q., Xu C., Wei B., An L. (2011). Self-assembled carbon-silicon carbonitride nano-composites: High-performance anode materials for li-thium-ion batteries. J. Mater. Chem..

[5] Bernard S., Miele P. (2014). ChemInform abstract: Ordered mesoporous polymer-derived ceramics and their processing into hierarchically porous boron nitride and silico-boron carbonitride monoliths. Cheminform.

[6] Zhao R., Shao G., Cao Y., An L., Xu C. (2016). Temperature sensor made of polymer-derived ceramics for high-temperature applications. Sens. Actuat. A—Phys..

[7] Cao F., Kim D., Li X., Feng C., Song Y. (2002). Synthesis of polyaluminocarbosilane and reaction mechanism study. J. Appl. Polym. Sci..

[8] Gou Y., Wang H., Jian K., Wang Y., Wang J., Song Y., Xie Z. (2016). Facile synthesis of melt-spinnable polyaluminocarbosilane using low-softening-point polycarbosilane for Si-C-Al-O fibers. J. Mater. Sci..

[9] Yu Y., Li X., Cao F. (2005). Synthesis and characterization of polyaluminocarbosilane. J. Mater. Sci. Lett..

[10] He G., Chen J., Chen L., Xia H., Zhang L. (2009). Method for preparing polyaluminocarbosilane. J. Appl. Polym. Sci..

[11] Xie Z., Gou Y. (2016). Polyaluminocarbosilane as precursor for aluminum-containing SiC fiber from oxygen-free sources. Ceram. Int..

[12] Duan Y., Mo G., Chen H., Liang Y., Cui Z., Yang X., Zhu S., Li Z., He L., Huang Q. (2019). Synthesis of polyaluminocarbosilane with low branching extent using liquid polysilacarbosilane and aluminum acetylacetonate by high-pressure method. Appl. Organomet. Chem..

[13] Yang J., Cheng X., Yu Y., Zhang Y. (2011). Quantitative determinations in the molecular structures of polyaluminocarbosilane. Polymer.

[14] Duan Y., Mo G., Huangfu Z., Liang Y., Cui Z., He L., Huang Z., Chai Z., Huang Q. (2019). Effects of aluminium content on the molecular structure and properties of polyaluminocarbosilane for SiC fibre fabrication. Ceram. Int..

[15] An L., Wang Y., Bharadwaj L., Zhang L., Fan Y., Jiang D., Sohn Y., Desai V., Kapat J., Chow L. (2004). Silicoaluminum carbonitride with anomalously high resistance to oxidation and hot corrosion. Adv. Eng. Mater..

[16] Wang Y., An L., Fan Y., Zhang L., Burton S., Gan Z. (2005). Oxidation of polymer-derived SiAlCN ceramics. J. Am. Ceram. Soc..

[17] Babonneau F., Sorarú G., Thorne K., Mackenzie J. (1991). Chemical characterization of Si-Al-C-O precursor and its pyrolysis. J. Am. Ceram. Soc..

[18] Li X., Edirisinghe M. (2004). Evolution of the ceramic structure during thermal degradation of a Si-Al-C-O precursor. Chem. Mater..

[19] Zheng C., Li X., Wang H., Zhao D., Hu T. (2008). Evolution of crystallization and its effects on properties during pyrolysis of Si-Al-C-(O) precursor fibers. J. Mater. Sci..

[20] Chen L., Zhang L., Cai Z., Yu Y., Gu H., Zhang L. (2008). Effects of oxidation curing and sintering additives on the formation of polymer-derived near-stoichiometric silicon carbide fibers. J. Am. Ceram. Soc..

[21] Mah T., Hecht N., Mccullum J., Hoenigman K.H., Katz A., Lipsitt H. (1984). Thermal stability of SiC fibres (Nicalon). J. Mater. Sci..

[22] Ichikawa H. (2016). Polymer-derived ceramic fibers, Annu. Rev. Mater. Res..

[23] Yu Y., Tang X., Li X. (2008). Characterization and microstructural evolution of SiC(OAl) fibers to SiC(Al) fibers derived from aluminum-containing polycarbosilane, Compos. Sci. Technol..

[24] Yao R., Feng Z., Chen L., Zhang Y. (2013). Effects of oxidation curing and Al atoms on the formation of near-stoichiometric freestanding SiC(Al) films derived from polyaluminocarbosilane (PACS). J. Eur. Ceram. Soc..

[25] Hesegawa Y., Okamura K. (1983). Synthesis of continuous silicon carbide fibre, Part 3 Pyrolysis process of polycarbosilane and structure of the products. J. Mater. Sci..

[26] Babonneau F., Sorarù G.D., Mackenzie J.D. (1990). ^29^Si MAS-NMR investigation of the conversion process of a polytitanocarbosilane into SiC-TiC ceramics. J. Mater. Sci..

[27] Sorarù G.D., Babonneau F., Mackenzie J.D. (1990). Structural evolutions from polycarbosilane to SiC ceramic. J. Mater. Sci..

[28] Ly H., Taylor R., Day R., Heatley F. (2001). Conversion of polycarbosilane (PCS) to SiC-based ceramic Part II. Pyrolysis and characterization. J. Mater. Sci..

[29] Porte L., Sartre A. (1989). Evidence for a silicon oxycarbide phase in the Nicalon silicon carbide fibre. J. Mater. Sci..

[30] Laffon C., Flank A., Lagarde P., Laridjani M., Olry P., Cotteret J., Dixmier J., Miquel J., Hommel H., Legrand A. (1989). Study of Nicalon-based ceramic fibres and powders by EXAFS spectrometry, X-ray diffractometry and some additional methods. J. Mater. Sci..

[31] Wen Q., Yu Z., Riedel R. (2020). The fate and role of in situ formed carbon in polymer-derived ceramics. Prog. Mater. Sci..

[32] Chen D., Mo G., Qian J., He L., Huang Q., Huang Z. (2020). Synthesis of cyano-polycarbosilane and investigation of its pyrolysis process. J. Eur. Ceram. Soc..

[33] Ferrari A.C., Robertson J. (2000). Interpretation of Raman spectra of disordered and amorphous carbon. Phys. Rev. B.

[34] Tuinstra F., Koenig J. (1970). Raman spectrum of graphite. J. Chem. Phys..

[35] Maruyama B., Ohuchi F. (1991). H_2_O catalysis of aluminum carbide formation in the aluminum-silicon carbide system. J. Mater. Res..

[36] Lihrmann J.M., Tirlocq J., Descamps P., Cambier F. (1999). Thermodynamics of the Al-O-C system and properties of SiC-AlN-Al_2_OC composites. J. Eur. Ceram. Soc..

[37] Yang L., Zhu H., Deng C., Cui P. (2014). Synthesis of Al_2_OC compound by carbothermal reduction process with boron oxide additive. China’s Refract.

[38] Inoue K., Yamaguchi A. (2003). Synthesis of Al_4_SiC_4_. J. Am. Ceram. Soc..

